# First report of *Toxoplasma gondii* seroprevalence in peafowls in Yunnan Province, Southwestern China

**DOI:** 10.1186/1756-3305-5-205

**Published:** 2012-09-19

**Authors:** Yi-Ming Tian, Fei-Yan Dai, Si-Yang Huang, Zu-Hong Deng, Gang Duan, Dong-Hui Zhou, Jian-Fa Yang, Ya-Biao Weng, Xing-Quan Zhu, Feng-Cai Zou

**Affiliations:** 1 State Key Laboratory of Veterinary Etiological Biology, Key Laboratory of Veterinary Parasitology of Gansu Province, Lanzhou Veterinary Research Institute, Chinese Academy of Agricultural Sciences, Lanzhou, Gansu Province, 730046, People’s Republic of China; 2 College of Animal Science and Technology, Yunnan Agricultural University, Kunming, Yunnan Province, 650201, People’s Republic of China; 3 College of Veterinary Medicine, South China Agricultural University, Guangzhou, Guangdong Province, 510642, People’s Republic of China; 4 Xishuangbanna Prefecture Center for Animal Disease Control and Prevention, Jinghong, Yunnan Province, 666100, People’s Republic of China; 5 School of Life Sciences, Yunnan University, Kunming, Yunnan Province, 650091, People’s Republic of China

## Abstract

**Background:**

*Toxoplasma gondii* is an intracellular protozoan parasite infecting almost all warm-blooded animals, including birds, with a worldwide distribution. Surveys of *T. gondii* infection in wild birds have been reported extensively in the world, but little is known of *T. gondii* infection in peafowls worldwide. This study was performed to determine the seroprevalence of *T. gondii* infection in peafowls in Yunnan Province, southwestern China.

**Methods:**

Sera from 277 peafowls, including 272 blue peafowls (*Pavo cristatus*) and 5 green peafowls (*Pavo muticus*) originated from two geographic areas in Yunnan Province were assayed for *T. gondii* antibodies using the modified agglutination test (MAT).

**Results:**

Specific *T. gondii* antibodies were detected in 35 of 277 (12.64%) peafowls (MAT titer ≥ 1:5). Seropositive birds were found in both species, 33 in 272 blue peafowls and 2 in 5 green peafowls. There was no significant difference in *T. gondii* seroprevalence between the adolescent birds (6.74%) and the adult birds (6.67%) (*P >* 0.05). The geographical origins of peafowls was found to be highly associated with *T. gondii* infection in the present study, a statistically significant difference in *T. gondii* seropositivity was observed between peafowls from Kunming (31.08%) and those from Xishuangbanna Dai Autonomous Prefecture (5.91%) (OR = 10.956, 95% CI = 1.632-73.545, *P* = 0.014). Statistical analyses showed that there were no significant interactions between ages and geographical origins of peafowls (*P* > 0.05).

**Conclusions:**

The results of the present survey indicated that infection of peafowls with *T. gondii* is widespread in Yunnan Province, which has significant public health concerns and implications for prevention and control of toxoplamosis in this province. To our knowledge, this is the first seroprevalence report of *T. gondii* infection in China’s southwestern Yunnan Province.

## Background

Toxoplasmosis is one of the most common parasitic zoonoses, caused by the obligate intracellular protozoan *Toxoplasma gondii,* which can infect almost all warm-blooded animals, including birds [1-5]. It has been estimated that approximately one third of the world population and 7.88% of population in China have been infected [2,6,7]. Toxoplasmosis is generally benign or associated with mild nonspecific clinical symptoms in most patients. However, blindness and mental retardation can be caused in congenitally infected children, and *T. gondii infection* is ranked as a leading cause of death in immuno-compromised individuals, especially in acquired immuno-deficiency syndrome patients [2,8].

Wild and domestic felids are the definitive hosts of this protozoan parasite, being able to excrete sporulated oocysts into the environment. Intermediate hosts such as humans or birds can become infected post-natally by ingesting tissue cysts from undercooked meat, consuming food or drink contaminated with oocysts, or ingesting oocysts from the environment accidentally [2,9]. Birds are important intermediate hosts of *T. gondii* and infection of birds with *T. gondii* is considered important epidemiologically because infection of ground-foraging birds with *T. gondii* can indicate soil contamination with oocysts, which also represent a source of infection for cats [10]. Surveys of *T. gondii* infection in wild birds have been reported extensively in the world, clinical cases have been reported and *T. gondii* is considered to be one of the causes of mortality in birds of different species [11-18].

The peafowls (*Pavo*) include two Asiatic species of flying birds in the genus *Pavo* of the pheasant family Phasianidae, best known for the male's extravagant display feathers. The blue peafowl (*Pavo cristatus*) is widely distributed naturally in the tropical forests of Southeast Asia, but the green peafowl (*P. muticus*) is only naturally distributed in Yunnan Province and Tibetan areas in China. Due to hunting and a reduction in extent and quality of habitat, the green peafowl is considered endangered on the International Union for Conservation of Nature (IUCN) Red List of Threatened Species [19], and is listed as Category I in the list of Key Protected Wildlife in China. In addition to its ornamental value, blue peafowl is a rare breeding bird domesticated for meat in some areas of China. Recent studies have identified a number of pathogens (such as avian influenza, avian pox) of potential conservation concern for this species [20-22], but such information still remains relatively limited.

Data on peafowl infection with *T. gondii* is limited in the world, to date only one survey has been conducted in Shanghai Zoological Garden in China in 2000 [23]. The objective of the present investigation was to determine the seroprevalence of *T. gondii* infection in peafowls in Yunnan Province, southwestern China, and the results obtained will provide base-line information on potential risk factors associated with infection and potential implications for public health.

## Methods

### The investigated regions

Yunnan Province is the most southwestern province of China, known for its richness in natural resources, covering approximately 394,000 square kilometers with a population of 45.7 million. The survey was conducted in two administrative divisions in the province, Kunming City and Xishuangbanna Dai Autonomous Prefecture (Banna for short). Kunming is the capital and the largest city in Yunnan Province, best known for its spring-like weather year around, the average temperature is around 15°C during winter and 24°C during summer. It has a mean annual rainfall of 1,010 mm, an annual sunshine period of 2,250 h, and an annual frost-free period of 230 d. Banna is located in the southern end of Yunnan Province, has a tropical climate, and an annual precipitation of 1200 mm. Its average annual temperature is 21°C with no frost period throughout the year.

### Ethics statement

The present study was approved by the Animal Ethics Committee of Lanzhou Veterinary Research Institute, Chinese Academy of Agricultural Sciences (Approval No: LVRIAEC2011-006). All birds were handled in strict accordance with good animal practice according to the Animal Ethics Procedures and Guidelines of the People's Republic of China.

### Blood samples

A total of 277 blood samples were obtained from the wing vein of peafowls between November 2011 and February 2012 in Yunnan Province. After clotting and centrifugation, serum was separated and stored at −20°C until further analysis. Whenever possible, data regarding species, geographic origin and age of each peafowl were collected. Birds included 272 blue peafowls and 5 green peafowls*,* among them 203 blue peafowls from Banna were farmed in extensive production systems for meat and were generally kept in small herds of 20–100 animals in wired chambers, and 74 peafowls (69 blue peafowls and 5 green peafowls) from the Kunming Zoo, as an ornamental bird, were all free-range fed on peafowl gardens. Ages of peafowls were classified into two categories according to their growth cycle: the adult birds (≥ 24 months, 45 birds sampled) and adolescent birds (≥ 5 months or < 24 months, 163 birds sampled) according to the general practice of raising peafowls. The management data were obtained before collecting serum samples through personal interviews with the workers on these gardens or zoo veterinarians.

### Serological examination

Sera were examined by the modified agglutination test (MAT) to detect antibodies against *T. gondii* as described previously [24-27]. Compared to other serologic methods, MAT is considered sensitive and specific for detecting *T. gondii* antibodies in many animals, including birds because the whole killed *T. gondii* tachyzoites were used as antigen and the addition of 2-mercapthetanol avoided the IgM-like substances that interfere with the specificity of the test [24]. At the beginning, sera were tested at 1:5 dilution to screen for the anti-*T. gondii* antibodies with MAT. Then, sera with positive or doubtful reactions were diluted two-fold starting at 1:5 dilution and assayed for *T. gondii* antibodies. A chicken serum sample obtained through experimental infection by *T. gondii* with a MAT antibody titer of 1:640 [28] was used as positive control. The serum sample of this chicken obtained before experimental infection by *T. gondii* was used as negative control.

### Statistical analysis

Differences in seroprevalence of infected peafowls between the age groups and among associated factors were analyzed using the binary logistic regression in SPSS (Release18.0 standard version, SPSS Inc., Chicago, Illinois) for Windows. A probability (*P*) value < 0.05 was considered as statistically significant between levels within factors and interactions. Odds-ratios (OR) with 95% confidence intervals based on likelihood ratio statistics are reported.

## Results and discussion

Thirty-five of the 277 (12.64%) peafowl serum samples were positive for *T. gondii* antibodies by MAT at the cut-off titer of 1:5, with titers of 1:5 in 17 samples (48.57%), 1:10 in 5 samples (14.29%), 1:20 in 4 samples (11.43%), 1:40 in 5 samples (14.29%) and 1:80 in 4 samples (11.43%). *T. gondii* infection was detected in both blue peafowls (12.13%) and green peafowls (2/5) (Table 1). Species of peafowl was not included in the statistical analysis due to the small number of green peafowls sampled. There was no significant difference in *T. gondii* seroprevalence between the adolescent birds (6.74%) and the adult birds (6.67%) (*P >* 0.05). Statistical analysis showed that peafowls originating from Kunming had significantly higher *T. gondii* seropositivity (31.08% of 74 samples) compared to the birds from Banna (5.91% of 203) (*P* = 0.014) (Table 1). There was no statistical interaction between ages and regions (*P* > 0.05).

**Table 1:** Seroprevalence of * Toxopalsma gondii * infection in peafowls in Yunnan Province, southwestern China by modified agglutination test (MAT)

**Factor**	**Category**	**No. tested**	**No. positive (%)**	**Antibody titers**				
				**1:5**	**1:10**	**1:20**	**1:40**	**1:80**
Species	Blue peafowl (*Pavo cristatus*)	272	33 (12.13)	16	5	4	4	4
	Green peafowl (*Pavo muticus*)	5	2 (40)	1	0	0	1	0
Age	Adolescent (>5 <24 months)	163	11 (6.74)	7	3	0	0	1
	Adult (≥24 months)	45	3 (6.67)	2	0	0	1	0
	Unknown	69	21 (30.43)	8	2	4	4	3
Region	Banna*	203	12 (5.91)	8	3	0	0	1
	Kunming	74	23 (31.08)	9	2	4	5	3
	Total	277	35 (12.64)	17	5	4	5	4

*Banna: abbreviation of Xishuangbanna Dai Autonomous Prefecture.

In the present study, most of the infected birds had low titers, though MAT is considered a sensitive and specific method for detecting *T. gondii* antibodies in avian species, because many reports and data on isolation of viable *T. gondii* were available with the MAT, using isolation of the parasite as a standard [2]. However, the cut-off titer of MAT for positive infection in birds is not determined, although occasionally, viable *T. gondii* has been isolated from poultry with a MAT titer of only 1:5 [2,11,29,30]. Therefore, we stated all titers in the present study (Table 1). There are limited studies on the infection of *T. gondii* in peafowls in the world, and only one survey of *T. gondii* in peafowls was conducted in Shanghai Zoological Garden in China in 2000 that utilized MAT, where only 1 of the 5 examined peafowls was found positive with a titer of 1:640, the survey was conducted 10 years ago with low sampling size and the management of birds is constantly changing, hygiene in general has improved in recent years, so no firm conclusion should be drawn from that early observation.

The association between risk factors and *T. gondii* seropositivity was analyzed. Age is not a crucial factor for *T. gondii* infection in the peafowl groups in this study because no significant difference in *T. gondii* seroprevalence was observed between the adolescent peafowls (6.74%) and the adult peafowls (6.67%) (OR = 0.849, 95% CI = 0.213-3.380, *P* = 0.817). Statistical analysis showed a significant difference in *T. gondii* seroprevalence according to geographical region, in that peafowls originating from Kunming had significantly higher seropositivity (31.08% of 74 samples) compared to the birds from Banna (5.91% of 203) (OR = 10.956, 95% CI = 1.632-73.545, *P* = 0.014), suggesting that region is a main risk factor associated with *T. gondii* seropositivity. Generally, warm and humid climates are favorable for the survival of *T. gondii* oocysts and the transmission of the parasite [2]. As previously described, there is no marked difference in the climate of the two surveyed regions. However, it should be noted that all peafowls from Kunming were free-range, fed on peafowl gardens in the zoos, but all peafowls from Banna were commercially raised in confinement in wired chambers, cats did not have access to the peafowl-housing area, but were known to defecate in the peafowl feed stored in open bins, and other felids such as bobcats and cougars kept captive in zoos may be potential shedders of *T. gondii* oocysts too [31]. Therefore, the feeding conditions and management of the peafowls and the infected felids in the living environment may be the main reasons attributed to this difference in different regions.

Little information was available on *T. gondii* seroprevalence in felids in Yunnan Province, our preliminary survey showed that the seroprevalence of *T. gondii* infection in stray cats in Yunnan was 26.79% (unpublished data), indicating a high risk as a source of *T. gondii* infection for animals and humans. It is estimated that millions of tourists visit the Kunming zoo every year and visitors can feed the free-range birds in the peafowl garden. Regular visitors and the extensive management of the peafowl garden in the Zoo may also increase the risk of infection with *T. gondii*.

In the present study, we investigated the seroprevalence of *T. gondii* infection in 277 peafowls from two regions in Yunnan Province, southeastern China between November 2011 and February 2012. The number of blue peafowls sampled was large enough for the findings to be conclusive, but the sample size of green peafowls was quite small, thus this study has some potential limitations in that the results of the present study may not reflect the actual *T. gondii* seroprevalence in green peafowls, in both species in other seasons, and in other regions of Yunnan Province. Also, information on the genders of the examined peafowls was not available, resulting in the lack of statistical analysis of this important factor. Therefore, further investigations of a large number of free-range or wild peafowls of both genders in a longer observation period and the isolation of *T. gondii* from infected peafowls will be considered in the future.

## Conclusions

The results of the present survey indicate that infection of peafowls with *T. gondii* is widespread in Yunnan Province, and the prevalene is highly associated with geographical regions, which raises significant public health concerns and has implications for the prevention and control of toxoplamosis in this province. Therefore, it is necessary for peafowl raisers, public health authorities, tourists, and zoo veterinarians to pay more attention to this problem. Comprehensive practical control approaches and measures, such as the improvement of feeding conditions and management of peafowl, the better management of stray dogs and cats, and the enhanced control of rodents to eliminate the sources of *T. gondii* in the environment to prevent *T. gondii* exposure to residents, should be executed.

## Competing interests

The authors declare that they have no competing interests.

## Authors’ contributions

FCZ and XQZ conceived and designed the study, and critically revised the manuscript. YMT, FYD, SYH, ZHD and GD performed the experiments, analysed the data and drafted the manuscript. DHZ, JFY and YBW helped in study design, study implementation and manuscript revision. All authors read and approved the final manuscript.
